# Genome-Wide Analysis of the Cinnamoyl-CoA Reductase (CCR) Gene Family and Its Involvement in Lignin Biosynthesis and Stress Responses in Six Tea Plant Cultivars

**DOI:** 10.3390/ijms27072957

**Published:** 2026-03-24

**Authors:** Ni Yang, Gui-Nan Li, Jia-Qi Zhang, Yuan Gao, Zhi-Hang Hu, Ai-Sheng Xiong, Jing Zhuang

**Affiliations:** 1 Tea Science Research Institute, College of Horticulture, Nanjing Agricultural University, Nanjing 211800, China; 2021204040@stu.njau.edu.cn (N.Y.); liguinan@stu.njau.edu.cn (G.-N.L.); 2024204064@stu.njau.edu.cn (J.-Q.Z.); 2022204007@stu.njau.edu.cn (Z.-H.H.); 2 State Key Laboratory of Crop Genetics & Germplasm Enhancement and Utilization, College of Horticulture, Nanjing Agricultural University, Nanjing 211800, China; 2021027@njau.edu.cn

**Keywords:** *Camellia sinensis*, *CCR*, lignin biosynthesis, abiotic stress, expression patterns

## Abstract

Cinnamoyl-CoA reductase (CCR) is the first rate-limiting enzyme in the lignin biosynthetic pathway in higher plants. It catalyzes the conversion of cinnamoyl-CoA into the corresponding cinnamaldehydes. Tea plant (*Camellia sinensis*) is a perennial woody species. Systematic identification and functional characterization of the *CCR* gene family in tea plants is still limited. In this study, 202 *CCR* genes were identified from six tea plant cultivars, and a significant expansion of the *CCR* gene family was observed during the domestication process from wild to cultivated tea plants. A total of 50 *CsCCR* genes were identified in the tea cultivar ‘Shuchazao’, and they were distributed across 13 chromosomes. Multiple sequence alignment revealed that the key catalytic motifs NWYCYGK and H-X-X-K were fully conserved in CsCCR1, CsCCR2, and CsCCR3. Phylogenetic analysis showed that CsCCR1/2/3 clustered with AtCCR1/2 and PtrCCR2, which were known to be involved in lignin biosynthesis. Transcriptome data analysis showed that *CsCCR3* exhibited significantly higher transcript abundance in stems than in young, mature, and old leaves. *CsCCRL9*, *CsCCRL33*, *CsCCRL34*, and *CsCCRL36* also showed relatively high expression levels in stem. RT-qPCR further confirmed the high expression of *CsCCR3* and *CsCCRL33* in stems. Furthermore, comparison of *CCR* members derived from tandem and segmental duplication in the tea cultivar ‘Shuchazao’ showed clear differences in *Ka/Ks* ratios, expression correlations, and the distribution of stress-responsive *cis*-acting elements. This study provides new insights into the expansion and duplication-related functional divergence of the *CCR* gene family in tea plant and identifies key candidate genes potentially involved in lignin biosynthesis and stress responses.

## 1. Introduction

Tea plant [*Camellia sinensis* (L.) O. Kuntze] is one of the most important beverage crops worldwide and is cultivated in more than 60 countries and regions [1,2,3]. The beverage produced from its tender shoots has significant cultural, economic, and health value [4,5]. Tea quality and flavor are closely related to the biosynthesis and accumulation of various secondary metabolites in young shoots, including flavonoids, amino acids, lignin, and terpenoids [6,7]. Previous studies have demonstrated a significant negative relationship between tea shoot tenderness and lignin content [8]. Lignin and flavonoid metabolic pathways share the carbon flux of the phenylpropanoid pathway, and their metabolic balance directly affects the accumulation of key quality-related compounds in tea plants [9,10]. Lignin deposition enhances plant resistance to various biotic and abiotic stresses, such as drought, salinity, and pathogen invasion, by reducing water loss and strengthening cell wall stability [11,12,13,14]. In tea plants, lignin plays important roles in tissue development, tea quality formation, and stress tolerance.

Lignin is the major phenolic polymer in the secondary cell wall of plants. Its biosynthesis is a typical branch of the phenylpropanoid metabolic pathway and has been extensively studied in various plant species [15,16]. This pathway involves a series of key enzymes and their products. Among them, cinnamoyl-CoA reductase (CCR) is the first rate-limiting enzyme in the lignin-specific branch. It catalyzes the conversion of p-coumaroyl-CoA, caffeoyl-CoA, feruloyl-CoA, 5-hydroxyferuloyl-CoA, and sinapoyl-CoA into their corresponding cinnamaldehydes [17,18]. The *CCR* gene family has been identified in many plant species, and the number of members varies greatly among different species [17,19]. The *CCR* gene family is generally divided into two categories: typical *CCR* and *CCR-like* members (*CCRL*). The NWYCYGK catalytic motif serves as a characteristic marker of typical CCR proteins [18,20]. The H-X-X-K motif was subsequently demonstrated to serve as a more stable and reliable marker for defining typical CCR, and its validity was confirmed in both poplar and rice [21]. The research results indicated that functional *CCRs* were usually highly expressed in lignified tissues, such as stems and roots [22]. In higher plants, inhibition of *CCR* expression leads to a corresponding decrease in lignin biosynthesis, disrupts cell wall structure, alters lignin monomer composition, and leads to the accumulation of abnormal phenolic intermediates in the cell wall [23,24]. In *Arabidopsis thaliana*, 11 *CCR* genes have been identified, including 2 *CCR* genes and 5 *CCR-like* genes reported [25,26]. In vitro kinetic analyses showed that AtCCR1 was approximately five times more efficient than AtCCR2 in catalyzing feruloyl-CoA and sinapoyl-CoA. *AtCCR1* was mainly expressed in lignified tissues, while *AtCCR2* was involved in stress and pathogen responses [25,26,27]. Suppression of *AtCCR1* expression markedly reduced lignin content and led to collapsed vascular bundles. The *cad-c cad-d ccr1* triple mutant retained only about 50% of the lignin level of the wild type and displayed severe developmental defects, including dwarfism and male sterility [27,28]. In wheat, a total of 115 *CCR* genes have been identified, including both *TaCCR* and *TaCCR-like* members. Among them, *TaCCR5-5* and *TaCCR6-1* were verified to be involved in lignin biosynthesis [19]. In *Liriodendron chinense*, 13 *LcCCR* genes have been identified. *LcCCR13* showed higher expression in stems than in other tissues, and its overexpression in transgenic tobacco markedly enhanced CCR enzymatic activity and lignin accumulation [17]. In pear, 31 *PbCCR* genes have been identified. *PbCCR1*, *PbCCR2*, and *PbCCR3* were closely associated with lignin deposition in fruit stone cells [18]. In addition to regulating lignin biosynthesis, *CCR* also plays an important role in plant responses to biotic and abiotic stresses. In *Sorghum bicolor*, the expression of *SbCCR2* was significantly induced after aphid feeding [29]. In rice, overexpression of *OsCCR10* increases lignin content in roots and significantly enhances drought tolerance [30]. These findings indicated that *CCR* genes played a central role in lignin biosynthesis, and in regulating cell wall structure and stress signaling pathways, contributing to plant adaptation and defense under diverse stresses.

The systematic identification and functional characterization of the *CCR* gene family remains limited in tea plants. In this study, *CCR* gene family members were identified and comparatively analyzed in the genomes of six tea cultivars, and their expansion patterns were investigated. Based on the chromosome-level genome assembly of ‘Shuchazao’, the molecular characteristics of the *CsCCR* family were systematically characterized by integrating phylogenetic relationships, synteny and duplication events, conserved motifs, promoter *cis*-acting elements, chromosomal distribution, *Ka/Ks* selective pressure, transcriptome data, and comparisons among different duplication types. This study helps elucidate the expansion pattern of the *CCR* gene family in tea plants and its functional divergence associated with gene duplication. It also provided important theoretical insights and genetic resources for future studies on lignin biosynthesis regulation and related traits improvement.

## 2. Results

### 2.1. Evolutionary Expansion Patterns of the CCR Gene Family in Large-Leaf and Small-Leaf Tea Cultivars

**Figure 1:**
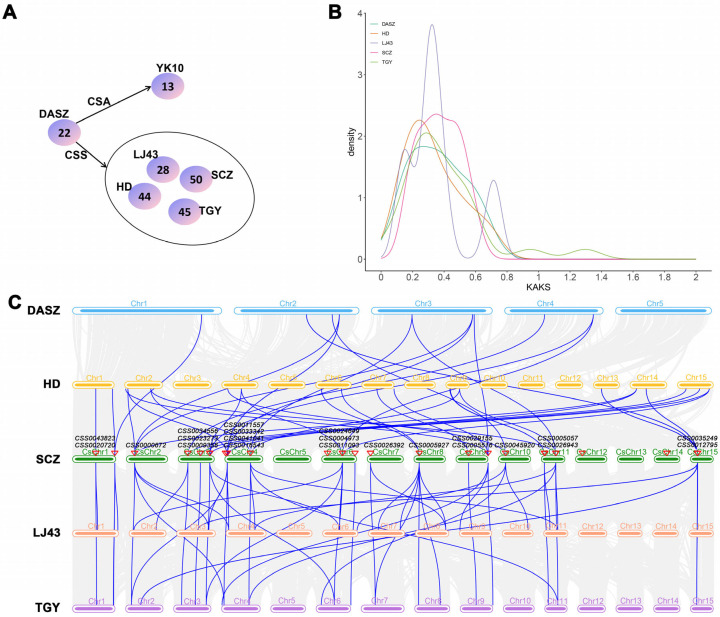
Identification of *CCR* genes in different tea cultivars. (**A**). Total number of *CCR* genes identified in different tea cultivars. (**B**). Density distribution of *Ka*/*Ks* ratios for *CCR* gene pairs, where the *x*-axis represents *Ka/Ks* values and the *y*-axis indicates the density. (**C**). Synteny analysis of the *CCR* gene family among tea cultivars DASZ, HD, LJ43, TGY, and SCZ. Background gray lines represent the syntenic regions identified between SCZ and the genomes of the other tea cultivars, whereas blue lines indicate the highlighted orthologous *CCR* gene pairs. DASZ, HD, LJ43, TGY, and SCZ represent different tea cultivars. Wild tea plant (DASZ), Yunkang 10 (YK10), Shuchazao (SCZ), Longjing 43 (LJ43), Tieguanyin (TGY), and Huangdan (HD).

To explore the potential differences in the evolution of *CCR* among different tea cultivars, this study selected a representative wild tea plant (DASZ), an Assamese variant (CSA-type) cultivated tea plant (Yunkang 10, YK10), and a Chinese small-leaf variant (CSS-type) cultivated tea plant [including Shuchazao (SCZ), Longjing 43 (LJ43), Tieguanyin (TGY), and Huangdan (HD)] for analysis. Whole-genome analysis revealed a significant expansion of the *CCR* gene family during tea domestication. DASZ contained 22 *CCR* genes, while YK10 had only 13. In CSS cultivars, the number of *CCR* genes increased substantially to 28~50, including SCZ (50), LJ43 (28), TGY (45), and HD (44) (Figure 1A and Table S1). These results indicated that *CCR* genes expanded significantly during the domestication process from wild to cultivated tea plants, which may be closely related to ecological adaptation and the evolution of domestication-related traits. Among the CSA- and CSS-type cultivated tea plants, ‘Shuchazao’ (SCZ) contained the largest number of *CCR* genes and was therefore selected for further detailed analysis.

To further reveal the driving mechanisms of *CCR* gene expansion in tea plants, MCScanX was used to analyze synteny relationships and gene duplication events of *CCR* genes (Table S2). The results showed that one, six, five, eight, and six syntenic blocks were identified in DASZ, SCZ, LJ43, TGY, and HD, respectively. Correspondingly, seven, fifteen, eight, nineteen, and fourteen tandemly duplicated gene pairs were detected in these genomes. The TGY cultivar contained 19 tandemly duplicated gene pairs, suggesting that tandem duplication played a predominant role in the expansion of the *CCR* gene family. To assess the selective pressures acting on duplicated *CCR* genes, *Ka*, *Ks*, and the *Ka/Ks* ratio were calculated for all identified gene pairs across five tea cultivars (Figure 1B and Table S2). The *Ka/Ks* ratios ranged from 0 to 1.297 across all tea cultivars, and the majority of duplicated gene pairs clustered between 0.25 and 0.55, indicating that the *CCR* gene family has predominantly evolved under purifying selection during evolution. One tandem duplicated gene pair in TGY showed a *Ka/Ks* value of 1.297. This elevated ratio was associated with an extremely low *Ks* value (0.022), possibly reflecting a recent gene duplication event or an estimation bias.

### 2.2. Syntenic Conservation and Orthologous Clustering Analysis of the CCR Gene Family Among Different Tea Cultivars

To elucidate the evolutionary relationships of *CCR* genes between wild and cultivated tea plants, the synteny analysis was performed among SCZ, DASZ, LJ43, HD, and TGY using the MCScanX tool (Figure 1C). The results showed that the *CCR* gene family exhibited strong syntenic conservation across all analyzed genomes. A total of 15 syntenic blocks were identified between SCZ and DASZ, and these regions were largely conserved in LJ43, HD, and TGY. The number of syntenic blocks among cultivated teas increased markedly, with 26, 29, and 31 blocks detected between SCZ and LJ43, HD, and TGY, respectively. This increasing trend reflected a higher retention of *CCR*-associated genomic segments in cultivated tea plants, suggesting that the *CCR* gene family may have undergone gradual expansion during the evolutionary transition from wild to cultivated tea.

**Figure 2:**
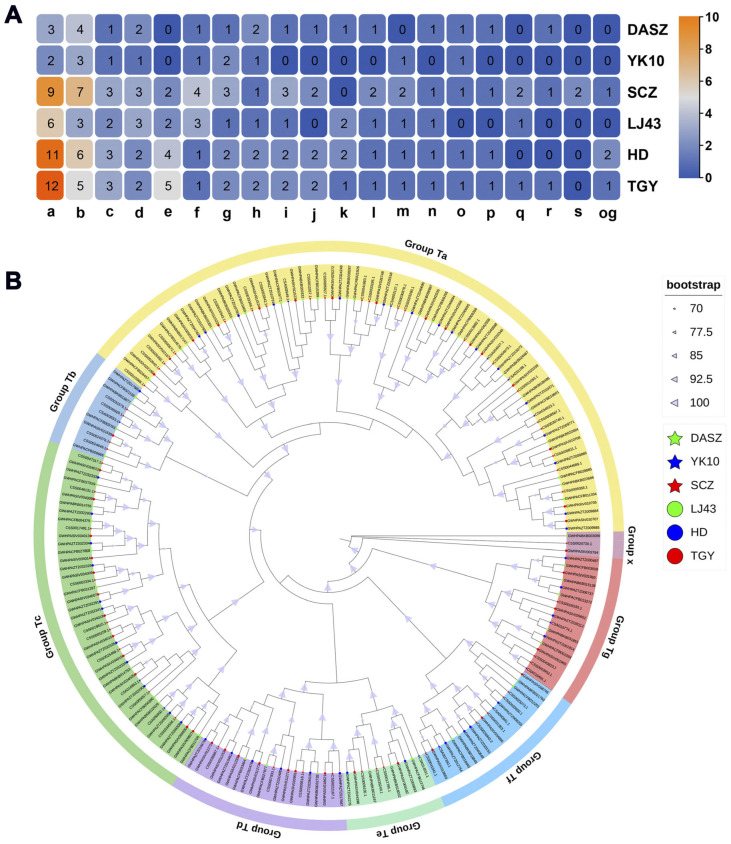
Orthologous grouping and phylogenetic analysis of the *CCR* gene family in different tea cultivars. (**A**). Orthologous grouping of the *CCR* gene family in different tea cultivars, with numbers indicating the gene counts in each group. (**B**). Phylogenetic tree constructed based on CCR proteins from different tea cultivars.

To investigate the distribution patterns of *CCR* family members in tea plants, the orthologous clustering analysis was conducted among different tea cultivars (Figure 2A). A total of 19 orthologous groups (a~s) and one outgroup were identified, among which groups a~c showed significant expansion in cultivated tea cultivars. In the SCZ cultivar, two genes (*CSS0012795* and *CSS0035249*) were uniquely retained in group s, while single-copy genes were observed in groups h, n, o, p, and r. A phylogenetic tree constructed by the maximum likelihood method classified *CCR* genes into seven evolutionary clades (Ta~Tg) and one outgroup (x) (Figure 2B). The Ta clade contained genes from orthologous groups b, i, n, p, q and og. The orthologous grouping results were highly consistent with the phylogenetic relationships, confirming the reliability of the analysis.

### 2.3. Identification, Chromosomal Localization, and Physicochemical Characterization of the CCR Gene Family in C. sinensis ‘Shuchazao’

**Figure 3:**
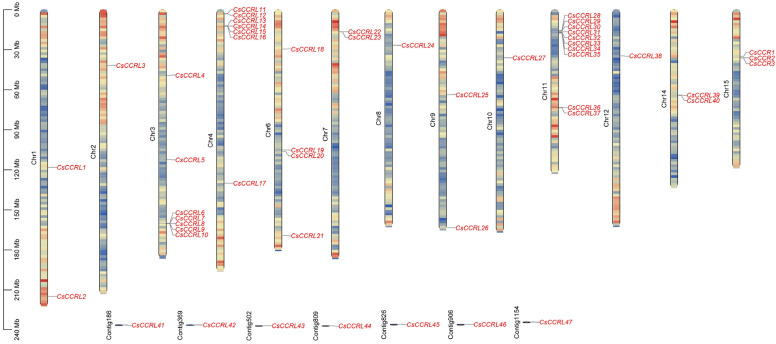
Chromosomal distribution of the *CsCCR* gene family. Each vertical bar represents a chromosome, and the scale on the left indicates the physical position in megabases (Mb). Gene density along each chromosome is shown as a heat map, using the gene density function in TBtools. Red and blue indicate regions with high and low gene density, respectively. *CsCCR* and *CsCCRL* denote cinnamoyl-CoA reductase and *CCR-like* genes in *C. sinensis*, respectively.

Based on the whole-genome sequence of the tea cultivar ‘Shuchazao’, a total of 50 cinnamoyl-CoA reductase (*CCR*) genes were systematically identified (Table S3). According to their chromosomal locations and the presence of the characteristic CCR motifs NWYCYGK and H-X-X-K, these genes were named *CsCCR1~CsCCR3* and *CsCCRL1~CsCCRL47*, respectively. Based on the conservation of these signature motifs, these genes were classified into two categories: typical *CsCCR* (*CsCCR1~CsCCR3*) and atypical *CsCCR* (*CsCCRL1~CsCCRL47*). The *CsCCR* genes were distributed across 13 chromosomes, with an additional seven members located on unanchored contig regions. *CsCCR1*, *CsCCR2*, and *CsCCR3* were located on chromosome 15 (Chr15). Chromosome 11 (Chr11) contained the largest number of *CsCCR* genes, with ten members (*CsCCRL28~CsCCRL37*) (Figure 3). The physicochemical property analysis showed that CsCCR proteins contained 246~368 amino acids, with molecular weights ranging from 27.10 to 40.80 kDa, isoelectric points (*pI*) from 5.03 to 8.84, and aliphatic indices between 75.41 and 102.86. It is noteworthy that eight CsCCR proteins exhibited instability index values greater than 40. Subcellular localization prediction indicated that all CsCCR proteins were localized in the cytoplasm (Table S3).

### 2.4. Multiple Sequence Alignment and Phylogenetic Analysis of the CsCCR Gene Family

**Figure 4:**
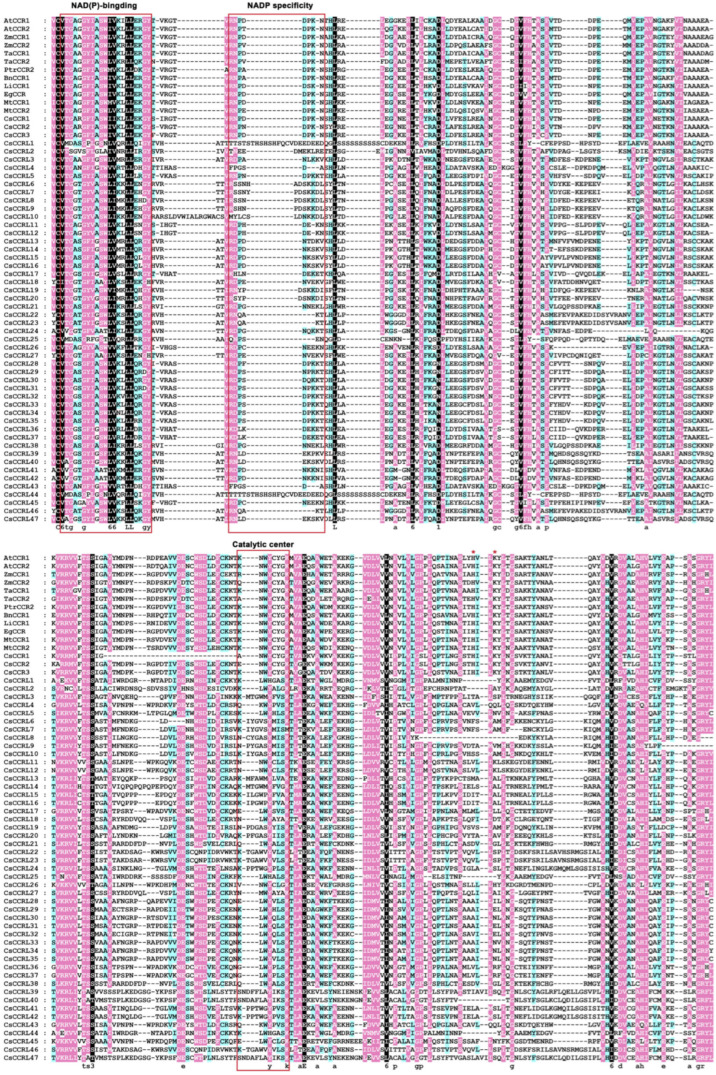
Multiple sequence alignment of CsCCR and CCR proteins from other plant species. Detailed gene information for CCR proteins from other plant species is provided in Table S4. The conserved NAD(P)-binding motif, the NADP-specific motif, and the catalytic signature motif NWYCYGK are marked with red boxes. Red asterisks (*) denote the H-X-X-K catalytic signature motif. Lowercase letters represent the consensus sequence. Background colors indicate different levels of sequence conservation.

**Figure 5:**
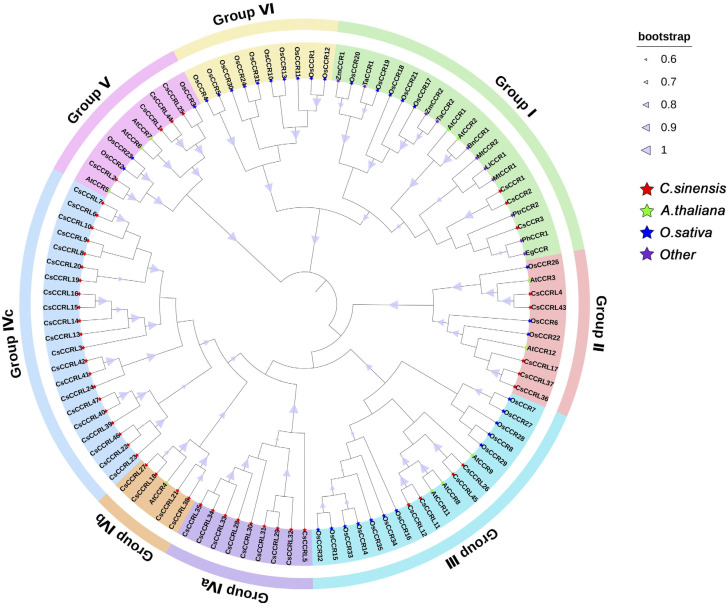
Phylogenetic tree of CsCCR and CCR proteins identified from other plant species. The phylogenetic tree was constructed using 105 CCR proteins, including 50 from *C*. *sinensis* (CsCCR), 11 from *A. thaliana* (AtCCR), 33 from *O. sativa* (OsCCR), 2 from *Z*. *mays* (ZmCCR), 2 from *T*. *aestivum* (TaCCR), 2 from *Medicago truncatula* (MtCCR), 1 from *P. trichocarpa* (PtrCCR2), 1 from *B*. *napus* (BnCCR1), 1 from *Leucaena leucocephala* (LlCCR1), 1 from *Eucalyptus gunnii* (EgCCR), and 1 from *Petunia × hybrida* (PhCCR). Detailed protein ID information is provided in Table S4.

Sequence alignment results showed that all CsCCR proteins were consistent with CCR proteins from other plant species, each containing the typical NAD(P)-binding site and conserved motifs related to substrate catalysis. It exhibited a highly conserved catalytic structure similar to *A*. *thaliana* AtCCR1/2 and *P. trichocarpa* PtrCCR2, whose functions were already well defined. Except for CsCCRL1, CsCCRL25, and CsCCRL44, the key CCR motif NWYCYGK appeared in the Y-X-X-X-K form in the remaining CsCCR members. Among them, CsCCR1, CsCCR2, and CsCCR3 retained the full NWYCYGK motif and also carried the H-X-X-K motif, suggesting that they may have possessed the catalytic structural features typical of functional CCR enzymes (Figure 4). Phylogenetic analysis showed that 105 CCR proteins from *C. sinensis*, *A*. *thaliana*, *O. sativa* and other species were divided into six evolutionary groups (Figure 5). Group I contained CsCCR1, CsCCR2, and CsCCR3, which clustered with AtCCR1/2 from *A*. *thaliana*, PtrCCR2 from *P. trichocarpa*, BnCCR1 from *Brassica napus*, TaCCR1/2 from *Triticum aestivum*, and ZmCCR1/2 from *Zea mays*. This clade was recognized as the typical CCR group closely associated with lignin biosynthesis. Group IV exhibited species-specific patterns. The IVa and IVc subgroups were tea-specific evolutionary branches, and this group contained only one *A*. *thaliana* member (AtCCR4). Group V contained four CsCCR proteins that clustered with AtCCR5~7 from *A*. *thaliana* and OsCCR2/3/23 from rice.

### 2.5. CsCCR Synteny Analysis

**Figure 6:**
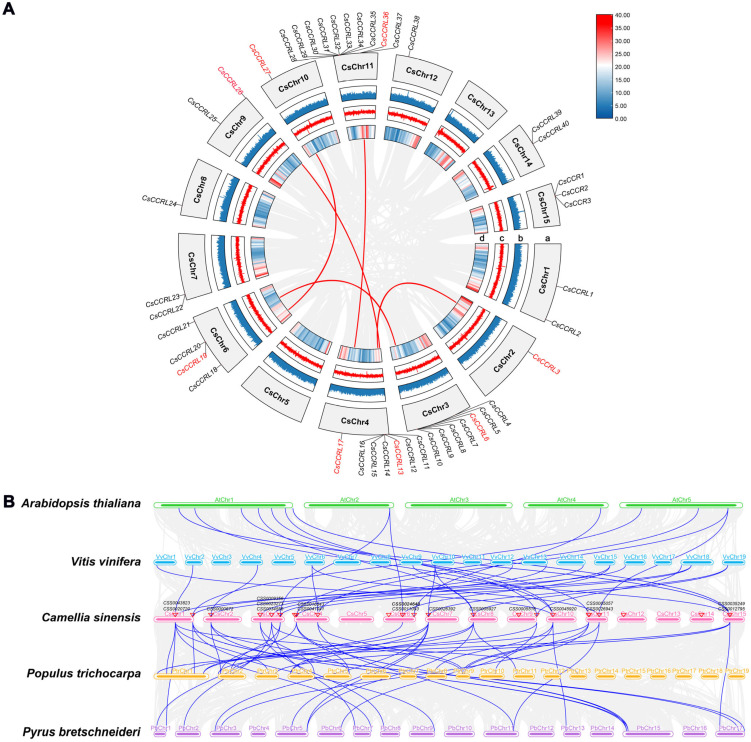
Synteny analysis of the *CsCCR* gene family. (**A**). Syntenic relationships of *CCR* genes within the genome of the tea cultivar ‘Shuchazao’. (**B**). Cross-species synteny analysis of *CsCCR* genes with *CCR* genes from *P*. *trichocarpa*, *P*. *bretschneideri*, *V*. *vinifera*, and *A*. *thaliana*. The red and blue connecting lines indicate syntenic *CCR* gene pairs. Genes highlighted in red indicate duplicated *CsCCR* genes.

To further validate the structural conservation of *CCR* members at the genomic level, synteny analysis was performed. In the tea cultivar ‘Shuchazao’, six conserved *CsCCR* syntenic blocks were identified (Figure 6A). The interspecies synteny analysis revealed 28, 23, 19, and 16 syntenic blocks of *CsCCR* genes between *C*. *sinensis* and *P*. *trichocarpa*, *P*. *bretschneideri*, *V*. *vinifera*. and *A*. *thaliana*, respectively. Among them, nine *CsCCR* genes, including *CSS0045920* and *CSS0012795*, maintained syntenic relationships across all four species (Figure 6B). Among them, CsCCR1 (*CSS0012795*) and CsCCR2 (*CSS0035249*) clustered with AtCCR1/2 and PtrCCR2 in the phylogenetic tree and showed collinearity with these known functional *CCR* genes at the genomic level (Figure 5 and Figure 6B).

### 2.6. Conserved Motif and Gene Structure Analysis of CsCCR Gene Family

**Figure 7:**
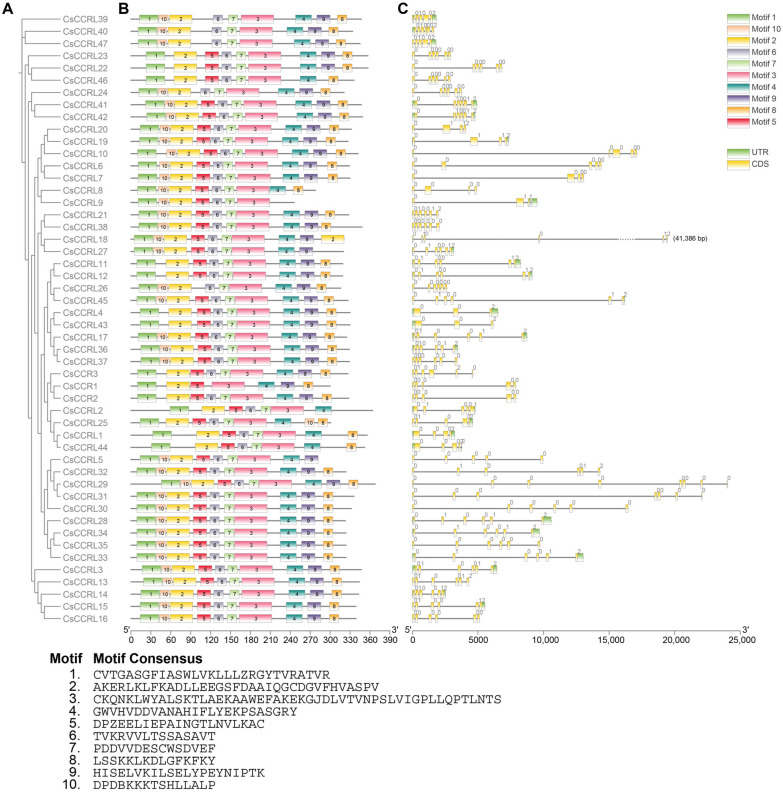
Gene structure and conserved motif analysis of CsCCR based on phylogenetic relationships. (**A**). Phylogenetic tree constructed based on the amino acid sequences of CsCCR proteins. (**B**). Distribution of conserved motifs in CsCCR proteins. Different colored blocks indicate distinct motifs. Motif 1 harbors the NAD(P)-binding site, whereas motif 3 contains the essential catalytic signature sequence NWYCYGK of CCR proteins. (**C**). Exon–intron structures of *CsCCR* genes. UTRs indicate untranslated regions, and CDSs indicate coding sequences.

Ten conserved motifs were identified in the *CsCCR* gene family of tea plants through MEME analysis. Motifs 1, 2, and 3 were highly conserved among all CsCCR members. Motif 1 contained the NAD(P)-binding site, while motif 3 harbored the catalytic signature sequence NWYCYGK, which was essential for CCR enzymatic activity. Motif 4 was found in all CsCCR proteins except CsCCRL9, whereas motif 5 was missing in four CsCCR members. Motifs 6 and 7 were conserved in all other CsCCR proteins except CsCCR1 (Figure 7B).

A systematic analysis was conducted to examine the exon-intron structures of the *CsCCR* gene family in tea plants. The results showed that the number of exons in tea *CsCCR* genes varied greatly (Figure 7C). The number of exons ranged from three to eight. *CsCCR1*, *CsCCR2*, *CsCCRL2*, *CsCCRL5*, *CsCCRL6*, *CsCCRL10*, *CsCCRL25*, *CsCCRL39*, and *CsCCRL47* contained five exons, which was consistent with the exon-intron patterns of functional *CCR* genes such as *AtCCR1*, *ZmCCR1*, and *SbCCR1*. Other structural variations were also observed in tea *CsCCR* genes. Thirty members contained six exons, six members contained four exons, three members contained only three exons, and one gene each contained seven and eight exons.

### 2.7. cis-Acting Element Analysis of CsCCR Gene Family in Tea Plant

**Figure 8:**
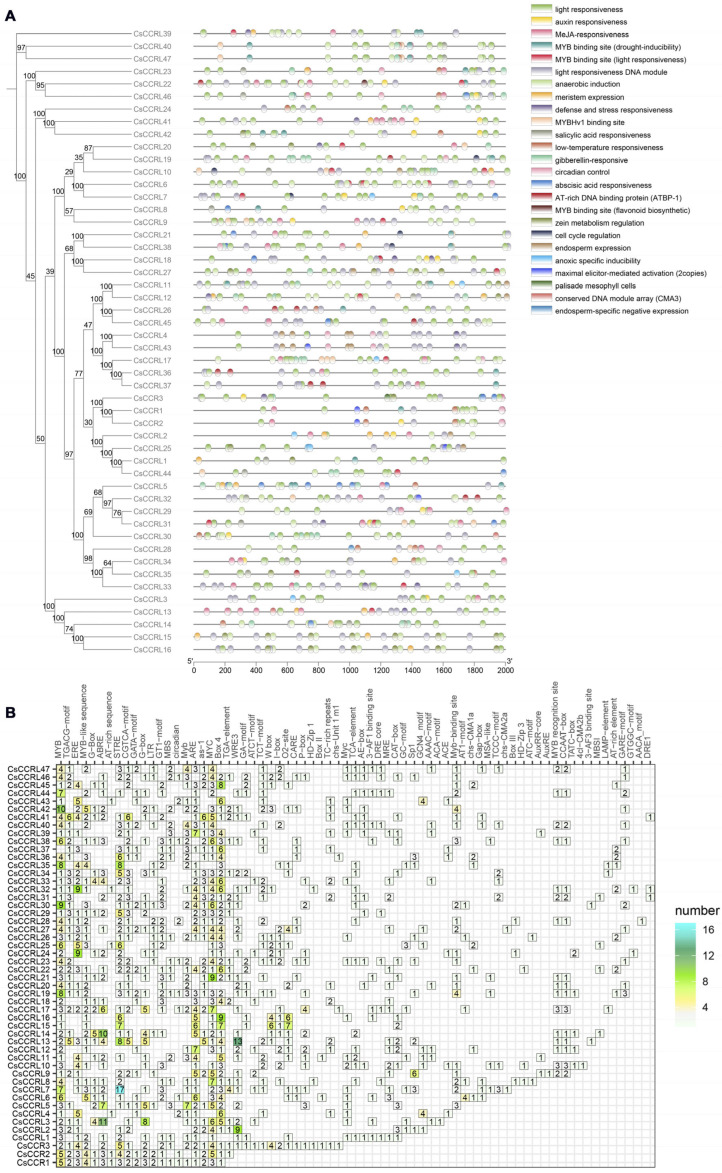
*cis*-acting regulatory element analysis of *CsCCR* gene promoters. (**A**). Spatial distribution of various *cis*-acting elements within the promoters of individual *CsCCR* genes. Different colored blocks indicate distinct *cis*-acting elements. (**B**). Statistical analysis of the number of *cis*-acting elements across individual *CsCCR* promoters.

To better understand the expression patterns and regulatory mechanisms of the *CsCCR* gene family, *cis*-acting elements located within the 2000 bp upstream promoter regions of 50 *CsCCR* genes were systematically analyzed (Figure 8). The results showed that the *CsCCR* gene promoters were enriched with various light-responsive elements, such as Box 4, G-box, GT1-motif and I-box, suggesting that their expression may be regulated by light signals. In addition, a wide range of hormone-responsive elements were identified in the promoters, such as auxin (TGA-element), gibberellin (TATC-box, P-box, GARE-motif), salicylic acid (TCA-element), abscisic acid (ABRE), and methyl jasmonate (CGTCA-motif, TGACG-motif). Among them, 45 *CsCCR* genes contained at least one hormone-responsive element, and 33 contained MeJA-responsive elements. Several stress-responsive elements were also identified, including low-temperature-responsive (LTR), anaerobic-induction (ARE), STRE, TC-rich repeats and GC-motif. Growth and development related elements were also detected, including circadian control elements (O2-site), meristem activity elements (CAT-box, GCN4-motif), vascular development elements (HD-Zip1) and cell cycle regulatory elements (MSA-like). Notably, multiple transcription factor binding sites such as MYB, W-box, MYC and MBS were widely distributed in the promoter regions.

### 2.8. Heatmap Analysis of Expression Patterns of CsCCR Gene Family in Tea Plant

**Figure 9:**
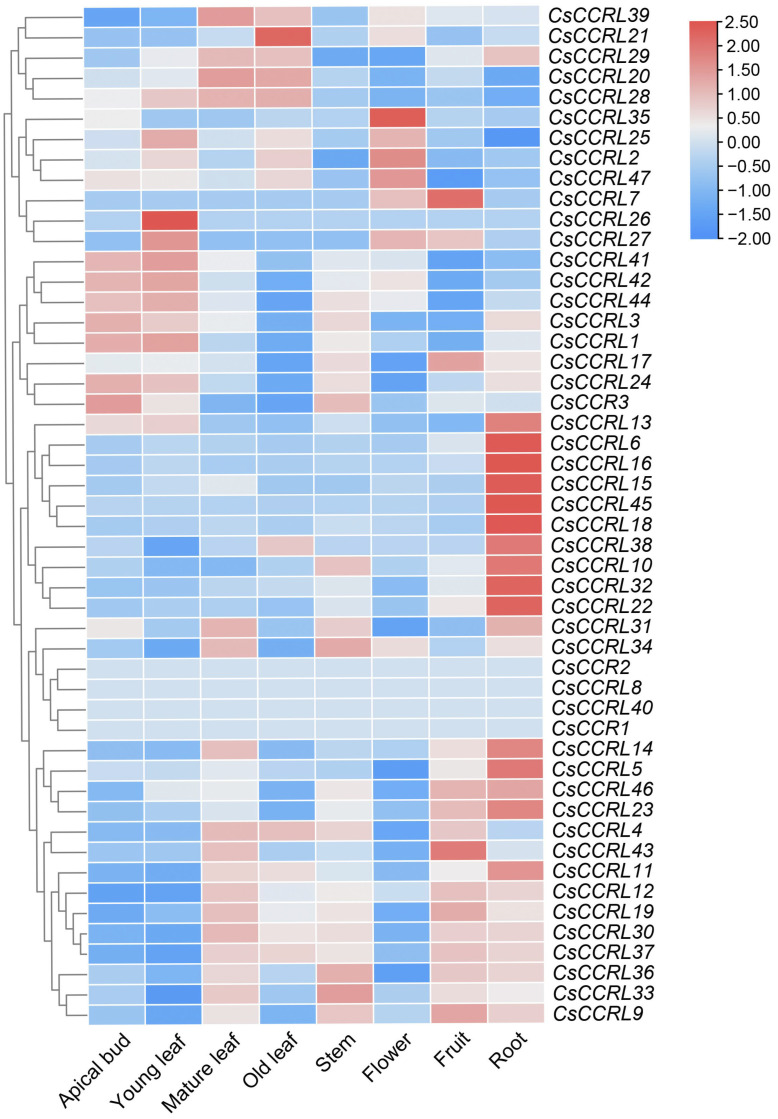
Tissue-specific expression heatmap of the *CsCCR* gene family. The horizontal axis represents different tissues and organs of the tea cultivar ‘Shuchazao’, and the vertical axis represents members of the *CsCCR* gene family. The color scale represents the transcriptional abundance of genes, with red indicating high expression and blue indicating low expression. The color scale on the right represents log_2_(TPM + 1) transformed values with row-wise scaling.

Based on the transcriptome data of *C. sinensis* ‘Shuchazao’, the Heatmap module in TBtools was used to visualize the expression patterns of *CsCCR* genes across different tissues and treatments. As presented in Figure 9, *CsCCR1*, *CsCCR2*, *CsCCRL8*, and *CsCCRL40* exhibited no detectable expression across all examined tissues, whereas the remaining genes showed expression in at least one tissue. Among these genes, *CsCCRL3* and *CsCCRL24* were highly expressed in buds, *CsCCRL1*, *CsCCRL25*, *CsCCRL26*, *CsCCRL41*, *CsCCRL42*, and *CsCCRL44* showed high expression in young leaves. Ten members, including *CsCCRL6*, *CsCCRL10*, and *CsCCRL13*, showed high expression levels in roots. *CsCCR3* showed much higher expression in buds and stems than in other tissues, while *CsCCRL33*, *CsCCRL34*, and *CsCCRL36* exhibited the highest transcript abundance in stems. *CsCCRL9* was highly expressed in fruits, followed by stems. *CsCCRL2*, *CsCCRL25*, *CsCCRL35*, and *CsCCRL47* showed relatively high transcript levels in flowers.

**Figure 10:**
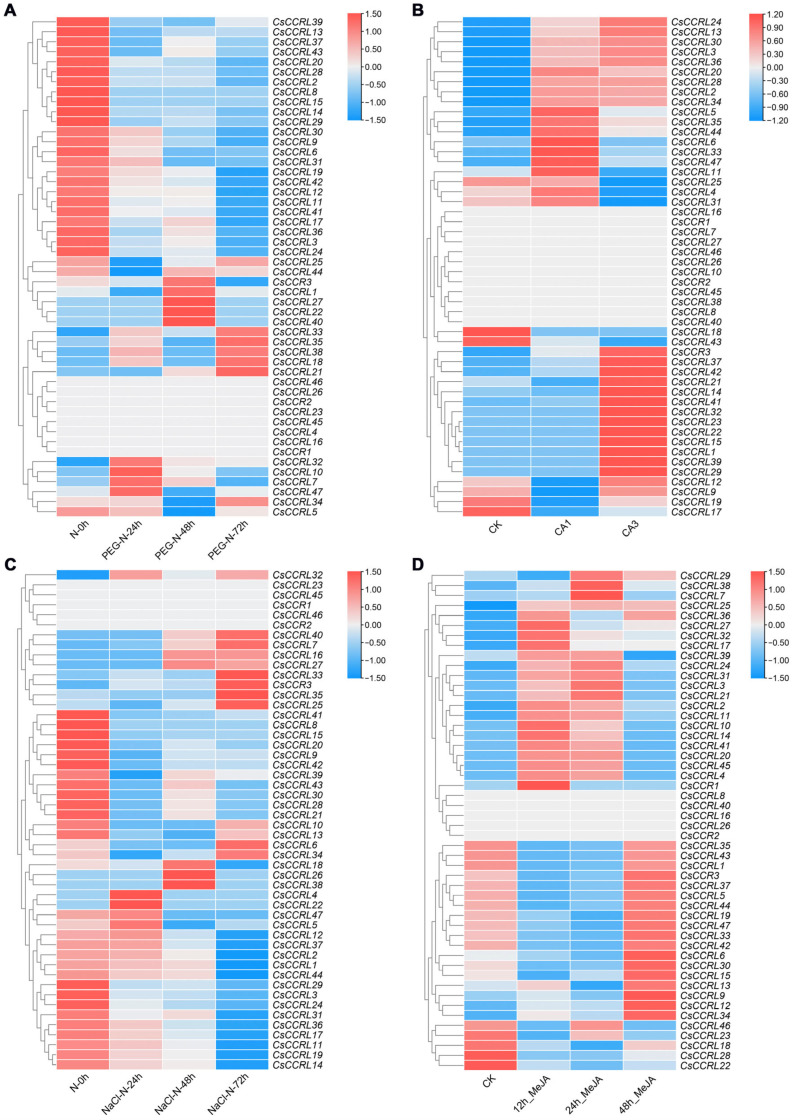
Heatmap of *CsCCR* gene expression under different treatments. (**A**). Drought treatment. (**B**). Cold treatment, including non-acclimation (CK), complete acclimation (CA1), and deacclimation (CA3). (**C**). Salt treatment. (**D**). MeJA treatment. The meaning of color scale is the same as in Figure 9. The color scale on the right represents log_2_(TPM + 1) transformed values with row-wise scaling.

Drought stress significantly upregulated the expression of *CsCCRL7*, *CsCCRL10*, *CsCCRL32*, and *CsCCRL47* at 24 h. At 48 h, the expression of six *CsCCR* genes, such as *CsCCR3* and *CsCCRL40*, was upregulated. At 72 h, the expression of *CsCCRL18*, *CsCCRL21*, *CsCCRL33*, *CsCCRL35*, and *CsCCRL38* reached their peak. *CsCCRL25* and *CsCCRL44* exhibited an expression pattern that was first inhibited and then activated (Figure 10A). After full cold acclimation, the expression of 18 *CsCCR* genes, including *CsCCRL31* and *CsCCRL33*, was markedly increased. During deacclimation, genes such as *CsCCR3*, *CsCCRL1*, and *CsCCRL12* were specifically activated (Figure 10B). Under salt stress for 72 h, eight *CsCCR* genes, including *CsCCR3*, *CsCCRL25*, *CsCCRL33*, and *CsCCRL34*, reached their highest expression levels. *CsCCRL18*, *CsCCRL26*, and *CsCCRL38* showed peak expression at 48 h (Figure 10C). Under exogenous MeJA treatment, several *CsCCR* genes showed varying degrees of response. Among them, *CsCCR3*, *CsCCRL5*, and *CsCCRL33* reached their highest expression levels at 48 h (Figure 10D).

### 2.9. Functional Divergence Driven by Different Gene Duplication Modes

**Figure 11:**
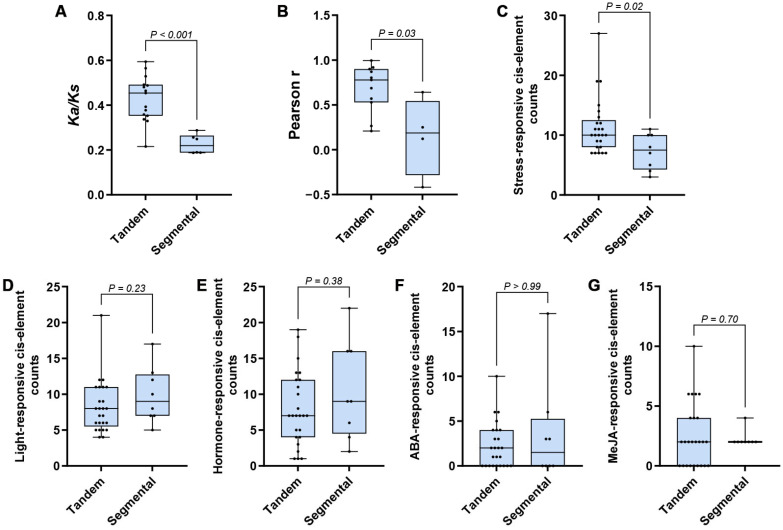
Functional divergence of expanded *CsCCR* genes driven by different duplication modes. (**A**). *Ka/Ks* ratio distributions of tandemly and segmentally duplicated gene pairs in the tea cultivar ‘Shuchazao’. (**B**). Distribution of tissue expression correlations (Pearson *r*) for tandemly and segmentally duplicated gene pairs in the tea cultivar ‘Shuchazao’. Transcriptome TPM values from eight tissues were used to calculate expression correlations. (**C**–**G**). *cis*-acting element counts in the promoters of tandem- and segmental-derived *CsCCR* genes. Stress-responsive *cis*-element counts (**C**), light-responsive *cis*-element counts (**D**), total hormone-responsive *cis*-element counts (**E**), ABA-responsive *cis*-element counts (**F**), and MeJA-responsive *cis*-element counts (**G**). A two-tailed Mann–Whitney U test was used to evaluate differences between groups, with significance defined as *p* < 0.05.

A total of 21 duplicated *CCR* gene pairs were identified in the tea cultivar ‘Shuchazao’, including 15 tandemly duplicated pairs and 6 segmentally duplicated pairs. Among these pairs, *CsCCR3* (*CSS0009155*) and *CsCCR1* (*CSS0012795*) constituted a tandem duplication event. The *Ka*, *Ks*, and *Ka/Ks* values were calculated for each duplicated gene pair. The results showed that the *Ka/Ks* ratios of all duplicated pairs were <1, suggesting that these duplicated genes were predominantly under purifying selection. Further comparison of *Ka/Ks* distributions between different duplication types revealed significant differences between tandemly and segmentally duplicated gene pairs (Figure 11A).

Based on transcriptome data, the Pearson correlation coefficient (*r*) of expression was calculated for each duplicated gene pair across eight tissues. The distributions of *r* values were then compared between different duplication types. The results showed significant differences in the distribution of expression correlations among duplicated gene pairs from different duplication modes. Segmentally duplicated gene pairs generally showed lower expression similarity, and some pairs exhibited low or even negative correlations (Figure 11B). To clearly present the tissue expression patterns of genes from different duplication types, separate tissue expression heatmaps were constructed for tandem-derived and segmental-derived members. The results indicated that the two groups displayed clear differences and heterogeneity in their relative expression patterns across tissues (Figure S1).

To further examine whether genes expanded through different duplication modes showed differences in *cis*-regulatory patterns, *cis*-acting elements in the promoter region (2 kb upstream) of each gene were functionally classified and compared based on counts per gene. The results showed that the number of stress-responsive *cis*-acting elements in the promoters of the tandem group was significantly higher than that in the segmental group (Figure 11C). In contrast, the total numbers of light-responsive, ABA-responsive, MeJA-responsive, and hormone-related elements did not differ significantly between the two groups (Figure 11D–G).

### 2.10. Spatiotemporal Expression Patterns and Stress-Responsive Characteristics of CsCCR Genes in Tea Plant

**Figure 12:**
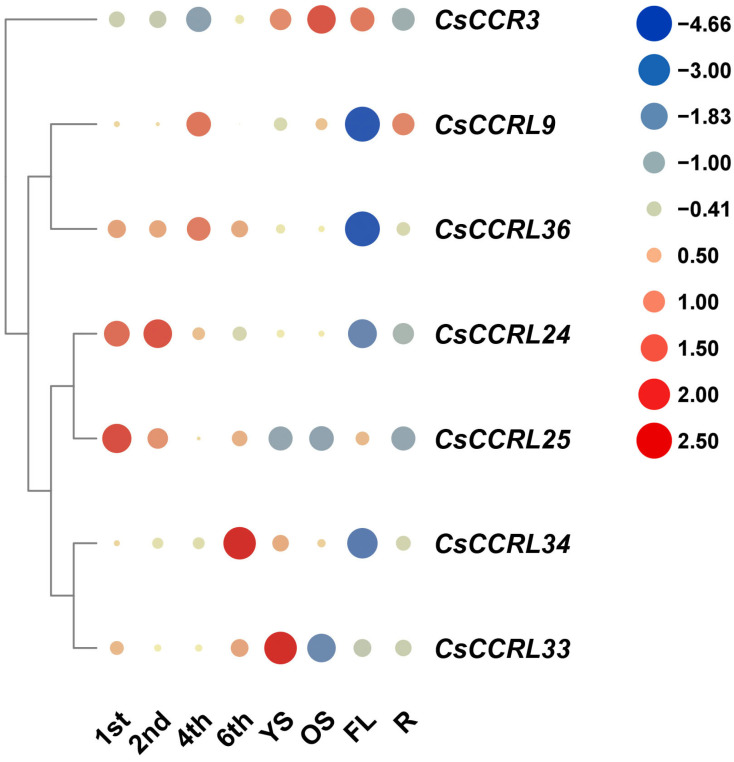
Spatiotemporal expression patterns of *CsCCR* genes. The horizontal axis indicates different tissues of tea cultivar ‘Shuchazao’ (1st: first leaf; 2nd: second leaf; 4th: fourth leaf; 6th: sixth leaf; YS: young stem; OS: old stem; FL: flower; R: root), and the vertical axis displays selected members of the *CsCCR* gene family. The color scale represents log_2_-transformed expression values, with the same meaning as in Figure 9.

To explore the potential roles of the *CsCCR* gene family in tea plant growth, development, and abiotic stress responses, highly expressed members (including *CsCCR3*, *CsCCRL9*, *CsCCRL24*, *CsCCRL25*, *CsCCRL33*, *CsCCRL34*, and *CsCCRL36*) were selected based on transcriptome data. Their expression patterns were further validated by RT-qPCR. The results showed that *CsCCRL24* and *CsCCRL25* were highly expressed in the first and second leaves, while *CsCCRL34* exhibited the highest expression in old leaves (the sixth leaf). *CsCCRL33* showed specific high expression in young stems, and *CsCCR3* was strongly expressed in old stems and flowers (Figure 12).

**Figure 13:**
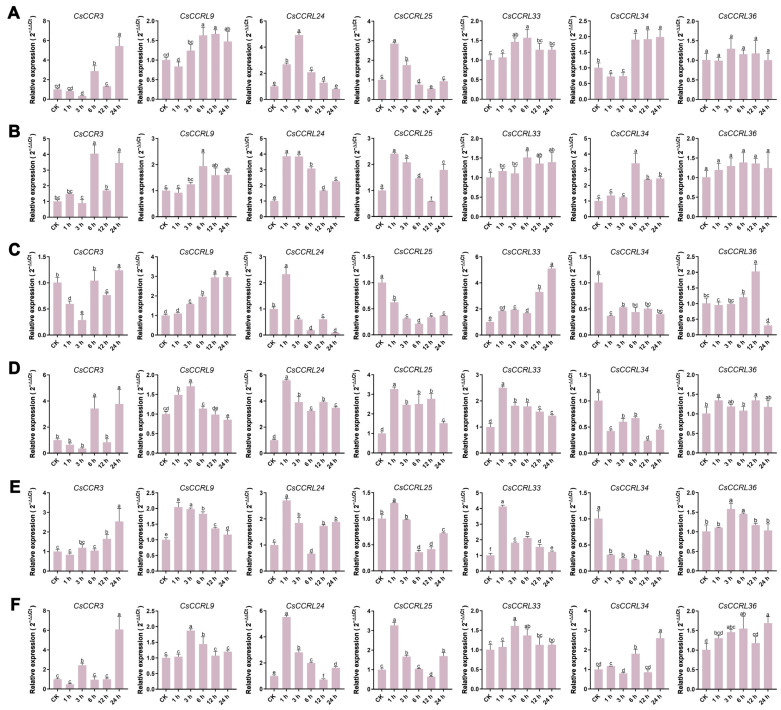
Expression patterns of *CsCCR* genes in tea plant under different abiotic stresses and hormone treatments. (**A**). Drought stress. (**B**). Salt stress. (**C**). High-temperature stress. (**D**). Low-temperature stress. (**E**). ABA treatment. (**F**). GA_3_ treatment. Different lowercase letters indicate statistically significant differences at *p* < 0.05 (one-way ANOVA).

Under drought stress, *CsCCR3* showed the highest expression at 24 h. *CsCCRL24* and *CsCCRL25* peaked at 3 h and 1 h, respectively, and then declined. *CsCCRL33* reached its highest level at 6 h. *CsCCRL9* and *CsCCRL34* showed a downregulation followed by recovery, with expression at 6 h, 12 h, and 24 h significantly higher than the control (Figure 13A). Under salt stress for 6 h, *CsCCR3*, *CsCCRL9*, *CsCCRL33*, and *CsCCRL34* showed the highest expression levels, while *CsCCRL24* and *CsCCRL25* showed rapid responses and reached their peak expression at 1 h (Figure 13B). Under heat treatment, *CsCCR3* was significantly upregulated at 24 h. *CsCCRL9* and *CsCCRL33* showed increasing expression over time. *CsCCRL24* was transiently upregulated at 1 h and then declined, while *CsCCRL25* was repressed (Figure 13C). Under cold stress, *CsCCR3* expression increased significantly at 6 h and 24 h. *CsCCRL24*, *CsCCRL25*, and *CsCCRL33* were significantly upregulated at all time points, while *CsCCRL9* reached its maximum expression at 3 h (Figure 13D). Both ABA and GA_3_ significantly induced the expression of *CsCCR3* at 24 h. Under ABA treatment, *CsCCRL9* and *CsCCRL33* were positively responsive at all time points, while GA_3_ induced their maximum expression at 3 h. *CsCCRL24* and *CsCCRL25* exhibited rapid responses to ABA and GA_3_, reaching their peaks at 1 h (Figure 13E,F).

## 3. Discussion

Cinnamoyl-CoA reductase (CCR) is a key rate-limiting enzyme in the biosynthesis of lignin monomers, and changes in its expression levels can alter lignin accumulation and structural composition [23]. Previous studies have reported that the tea plant genome experienced a whole-genome duplication (WGD) event. This event, together with subsequent large-segment duplications, promoted the expansion of secondary metabolism-related gene families such as flavonoids and terpenoids, and it also increased the number of genes associated with tea quality and stress resistance [4,31]. Gene duplication events, including WGD/segmental duplication and tandem duplication, were widely regarded as key drivers of gene family expansion and functional diversification in plants [32,33,34]. In this study, the number of *CCR* genes differed markedly between wild and cultivated tea plants. Wild tea plants contained 22 *CCR* genes, whereas CSS-type cultivated varieties contained 28~50 *CCR* genes, suggesting expansion of the *CCR* gene family during domestication. Synteny and duplication pattern analyses indicated that the expansion of the *CCR* gene family was driven by both WGD/segmental duplication and tandem duplication. Similar expansion patterns have been reported in *A. thaliana*, poplar, and rice [35]. The expansion of the *CCR* gene family may have been associated with domestication-related improvements in growth traits, differences in mechanical tissue structure, and environmental adaptation. It may also have been influenced by genome duplication history and other evolutionary processes. Future studies integrating population genetic selection signals with lignin-related trait data will be required to further validate these hypotheses.

Gene duplication increased gene copy number and often led to regulatory divergence after duplication, resulting in subfunctionalization or neofunctionalization [36]. In this study, most duplicated gene pairs across different tea cultivars showed *Ka/Ks* ratios < 1, suggesting that the *CCR* gene family was predominantly under purifying selection. This pattern was consistent with the functional conservation of key enzymes involved in lignin biosynthesis. Further comparison in ‘Shuchazao’ revealed significant differences in *Ka/Ks* distributions between tandem and segmental duplicated pairs, suggesting that genes generated by different duplication mechanisms experienced different levels of evolutionary constraint. At the expression level, the distribution of expression correlations also differed significantly between the two duplication types. *CsCCR* members derived from different duplication events displayed heterogeneous expression patterns across eight tea tissues, suggesting that functional differentiation may arise through reallocation of tissue-specific expression. Promoter analysis revealed that tandem duplicates contained significantly more stress-responsive elements than segmental duplicates, whereas other regulatory modules showed no significant differences. These results suggested that regulatory divergence after duplication was concentrated in stress-related pathways. Overall, *CsCCR* family expansion likely resulted in functional specialization rather than simple redundancy.

Multiple sequence alignment showed that CsCCR1, CsCCR2, and CsCCR3 retained the typical CCR catalytic motifs NWYCYGK and H-X-X-K. Phylogenetic analysis further showed that they clustered in the same branch as AtCCR1/2, PtrCCR2, and ZmCCR1/2, which have been previously reported to be involved in lignin biosynthesis [27,37,38,39]. These findings suggested that these genes likely represent the evolutionarily conserved canonical *CCR* lineage in angiosperms. Cross-species synteny analysis revealed multiple conserved *CCR* syntenic blocks between tea plant and other species, including *A. thaliana*, grape, pear, and poplar. *CsCCR1/2* showed conserved syntenic relationships with *AtCCR1/2* and *PtrCCR2*. Gene structure analysis further indicated that nine *CsCCR* genes possessed the typical five-exon structure, similar to functional *CCR* genes reported (*AtCCR1*, *EuCCR*, *ZmCCR1*, and *SbCCR1*) in other plant species [37,38,40]. Although *CsCCR3* contained six exons, its overall gene structure closely resembled the typical *CCR* structure. These results supported the evolutionary conservation and functional constraint of the core *CCR* lineage in angiosperms.

Transcriptome analysis showed that *CsCCR3* exhibited significantly higher transcript abundance in buds and stems compared with other tissues. *CsCCRL33*, *CsCCRL34*, and *CsCCRL36* displayed elevated transcript levels in stem tissues. RT-qPCR analysis further confirmed that *CsCCR3* showed significantly higher expression in young and mature stems than in leaves. *CsCCRL33* was specifically highly expressed in young stems. This expression pattern was consistent with typical *CCR* genes such as *AtCCR1*, *OsCCR20*, and *ZmCCR1*, which are highly expressed in lignified tissues [25,27,38,40]. These findings suggested that *CsCCR3* likely represents a key candidate gene involved in lignin biosynthesis in tea plant. Considering that both *PbCCR1* and *PbCCR2* in pear participate in lignin biosynthesis with partial functional redundancy, and that *CCR-like* members in rice have also been shown to promote lignin deposition [30,41]. In this study, *CCR-like* genes such as *CsCCRL33* showed high expression in stems, suggesting that they may participate in cell wall lignification through redundant or compensatory mechanisms, or contribute to functional specialization in specific tissues or developmental stages. However, in vitro enzyme assays and genetic functional validation were not performed in the present study. Future studies involving overexpression or knockout experiments, in vitro enzyme assays, and analyses of lignin content and composition will be required to verify their catalytic activity and biological functions. This study also found that *CsCCR1*, *CsCCR2*, and several members such as *CsCCRL8* and *CsCCRL40* showed no detectable expression across the analyzed tissues. Although *CsCCR1* and *CsCCR2* retained the complete catalytic motifs and belonged to the canonical *CCR* lineage, their transcripts were not detected. This pattern may be associated with promoter-mediated repression or epigenetic regulation, such as DNA methylation or chromatin state variation. Further studies using promoter activity assays and analyses of DNA methylation or chromatin accessibility may help clarify their silencing mechanisms and potential functions.

Promoter *cis*-acting elements play a key role in regulating gene transcription. In this study, the promoters of *CsCCR* genes in tea plants contained abundant light, hormone, stress and development related elements. Binding sites for transcription factors such as MYB, NAC, MYC, and W-box were widely present. The ABRE element activates stress-responsive genes through the SnRK2-AREB/ABF signaling cascade [42]. Previous studies showed that PbrMYB24 in pear and MYB46/83 and MYB58/63 in Arabidopsis activated lignin biosynthetic genes by binding to AC or SMRE elements [43,44,45]. In rice, OsNAC5 directly binds to the promoter of *OsCCR10*, leading to increased lignin deposition and improved drought resistance [30]. Based on the promoter analysis in this study, a similar transcriptional regulatory network may exist in tea plant. These transcription factors may interact with *CsCCR* promoters to regulate lignin biosynthesis, hormone signaling pathways, and responses to abiotic stress. Expression analysis showed that *CsCCRL24* and *CsCCRL25* responded rapidly to drought, salt, ABA, GA_3_ treatments, while *CsCCR3* and *CsCCRL33* were consistently upregulated under drought and ABA treatments. *CsCCRL34* was induced at the late stage of drought stress but showed a different pattern from ABA treatment, suggesting that it may respond to stress through an ABA-independent pathway. These differences may be associated with variation in promoter *cis*-element composition and regulatory divergence following gene duplication, which may contribute to functional specialization of *CsCCR* family members in cell wall formation and environmental adaptation. From the perspective of domestication and phenotypic adaptation, cultivated tea plants generally exhibited more stable growth traits and more developed mechanical tissues. The pattern of lignin deposition may be closely related to traits such as branch strength, cell wall construction, and the texture of young shoots. This study revealed an expansion of the *CCR* gene family in cultivated tea plants, and members derived from different duplication types showed differences in stress-related *cis*-elements. This variation may provide regulatory redundancy and flexibility for the spatial and temporal regulation of lignin deposition in tea plants. These features may have contributed to structural strengthening and stress adaptation during tea domestication. Future studies should integrate lignin content and monomer composition analysis, physiological measurements, and genetic validation of key *CsCCR* genes. These approaches will help clarify the roles of *CCR* genes in lignin biosynthesis and environmental adaptation in tea plants.

## 4. Materials and Methods

### 4.1. Plant Materials and Growth Conditions

Tea plants (*C. sinensis* cv. ‘Shuchazao’) used in this study were approximately two-year-old cuttings. Leaves at different developmental stages (first, second, fourth, and sixth leaves counted from the apical bud), young stems (non-lignified stems located at the upper part of the shoots), old stems (fully lignified stems), flowers (full-bloom stage; entire flowers), and roots (fine roots, with a pale or white appearance) were collected for tissue-specific expression analysis. All tissue samples were collected in the morning in October. Uniformly grown tea cuttings were treated with four abiotic stresses: low temperature (4 °C), high temperature (38 °C), drought (20% PEG-6000), and salt stress (200 mM NaCl). Two hormone treatments were also performed: 1 mM GA_3_ and 50 mg·L^−1^ ABA. Samples were collected at 0, 1, 3, 6, 12, 24 h for all treatments, with three biological replicates per time point [46,47]. All samples were immediately frozen in liquid nitrogen and stored at −80 °C for later analysis. The tea cuttings were cultivated in an artificial climate chamber at the State Key Laboratory of Crop Genetics & Germplasm Enhancement and Utilization of Nanjing Agricultural University (32°02′ N, 118°50′ E, Nanjing, China). The growth conditions were set as follows: photoperiod of 16 h light/8 h dark cycle, day and night temperature of 25 °C/18 °C, and relative humidity of 70% ± 5%.

### 4.2. Genome-Wide Identification and Characterization of CCR Genes in Tea Plants

Genome, protein, and CDS sequences, together with their annotation files, were downloaded for multiple plant species from several public databases. Data for tea cultivars, including wild tea plant (DASZ), Yunkang 10 (YK10), Shuchazao (SCZ), Longjing 43 (LJ43), Tieguanyin (TGY), and Huangdan (HD), were obtained from TPIA (http://tpia.teaplants.cn/index.html, accessed on 6 February 2025). The genome data of *Arabidopsis thaliana* were downloaded from TAIR (https://www.arabidopsis.org/, accessed on 6 February 2025), *Vitis vinifera* from Ensembl Plants (https://plants.ensembl.org/index.html, accessed on 6 February 2025), *Populus trichocarpa* from Phytozome v13 (https://phytozome-next.jgi.doe.gov/, accessed on 6 February 2025), and *Pyrus bretschneideri* from GigaDB (https://gigadb.org/dataset/100083, accessed on 6 February 2025). Previously identified CCR protein sequences from other species were collected from their respective databases.

The *CCR* gene family was identified based on two methods. BLASTP searches (E-value < 1 × 10^−5^) were conducted using known *A*. *thaliana* CCR proteins as queries against the tea plant genomes. Hidden Markov Models were built using confirmed CCR proteins from other plant species and the Pfam CCR domain (PF01370) as seed models for Simple HMM search (E-value < 1 × 10^−5^). The candidate sequences obtained by the two methods were deduplicated to obtain a preliminary set of *CCR* gene family members. To verify the reliability and accuracy of the *CCR* gene family members, the candidate sequences were domain-verified using the NCBI Conserved Domain Database (CDD). Sequences lacking the conserved NAD(P)-binding motif were deleted to finalize the tea plant *CCR* gene family members.

### 4.3. Homology-Based Clustering and Phylogenetic Analysis of the CCR Gene Family

The CCR genes from different tea cultivars were clustered into orthogroups using OrthoFinder v2.5.5. Sequence similarity searches were performed internally by OrthoFinder v2.5.5, and the resulting similarity scores were normalized based on gene length and phylogenetic distance. The resulting sequence similarity graph was clustered using the Markov Cluster (MCL) algorithm to identify orthogroups. The number of CCR genes in each orthogroup was then counted for each tea cultivar.

Multiple sequence alignment of CCR gene family proteins from different tea cultivars was performed using the MUSCLE Wrapper implemented in TBtools-II v2.441. Poorly aligned regions were trimmed using the trimAl Wrapper in the same software. Phylogenetic analysis was then conducted using the IQ-TREE Wrapper in TBtools-II v2.441 based on the Maximum Likelihood (ML) method, with the optimal substitution model automatically selected under the default ‘Model: Auto’ setting. Branch support was assessed with 1000 ultrafast bootstrap replicates [48]. iTOL v7 (https://itol.embl.de/, accessed on 10 December 2025) was used to visualize and beautify the phylogenetic tree.

### 4.4. Chromosomal Distribution, Sequence Alignment, and Synteny Analysis of CsCCR Genes

Based on chromosome-level genome annotation data for the tea cultivar ‘Shuchazao’, the chromosomal localization of the CsCCR genes was mapped using TBtools. Multiple sequence alignment of CCR proteins from C. sinensis, A. thaliana, O. sativa and other species was performed using ClustalW as implemented in MEGA11 v11.0.10, and the alignment was visualized with GeneDoc v2.7. The aligned sequences were then imported into MEGA11 v11.0.10 for phylogenetic analysis. The phylogenetic tree was constructed using the Maximum Likelihood (ML) method under the LG + G substitution model, with 1000 bootstrap replicates to assess branch support. The phylogenetic tree was visualized and beautified using iTOL v7 (https://itol.embl.de/, accessed on 10 December 2025).

The One Step MCScanX-Super Fast module in TBtools was used to perform synteny analysis of tea plant genomes and to identify orthologous relationships with *A*. *thaliana*, *V. vinifera*, *P. trichocarpa*, and *P. bretschneideri*. Orthologous and syntenic gene pairs were identified accordingly. The Multiple Synteny Plot and Advanced Circos tools were used to visualize the syntenic blocks. The Simple *Ka/Ks* Calculator (NG) was further used to calculate the nonsynonymous (*Ka*) and synonymous (*Ks*) substitution rates to evaluate the selective pressure during gene evolution.

### 4.5. Structural Characterization, Conserved Motif Identification, and Promoter cis-Element Analysis

Based on the whole-genome annotation data of ‘Shuchazao’, the gene structure of the *CsCCR* gene family was mapped. Conserved motifs were predicted by MEME Suite v5.5.7 online platform (https://meme-suite.org/meme/tools/meme, accessed on 6 April 2025) with the number of motifs set to 10 (E-value < 10^−5^). *cis*-acting elements were further identified in the promoter regions (2000 bp upstream of the transcription start site) of *CsCCR* gene family members through the PlantCARE database (https://bioinformatics.psb.ugent.be/webtools/plantcare/html/, accessed on 7 April 2025). All analysis results were visualized using TBtools software (Version: 2.441) [48].

### 4.6. Expression Profiling of the CsCCR Gene Family in Tea Plant Based on Heatmap Analysis

Transcriptome data of the tea cultivar ‘Shuchazao’ were obtained from TPIA (https://tpia.teaplants.cn/, accessed on 7 April 2025). The data included tissue-specific expression (apical buds, young leaves, mature leaves, old leaves, stems, flowers, fruits, and roots), responses to abiotic stresses (drought, salt, and cold), and MeJA treatment [3,31]. Transcript abundance was normalized as TPM (Transcripts Per Million) values and further log_2_(TPM + 1) transformed. The clustered heatmap of *CsCCR* gene family members was generated using the HeatMap Illustrator module in TBtools [48].

### 4.7. Analysis of Functional Divergence Driven by Different Duplication Modes

In the tea cultivar ‘Shuchazao’, duplicated *CsCCR* gene pairs identified by MCScanX were classified into tandem duplication and segmental duplication according to their duplication types. The *Ka*, *Ks*, and *Ka/Ks* values for duplicated gene pairs were obtained using the Simple *Ka/Ks* Calculator (NG) module in TBtools. Duplicated gene pairs were used as the statistical units to compare the *Ka/Ks* distribution differences between tandem and segmental duplication pairs.

Transcriptome data described in Section 4.6 were used to obtain the expression levels of each duplicated gene in eight tissues. For each duplicated gene pair, the Pearson correlation coefficient (*r*) of their cross-tissue expression vectors was calculated using GraphPad Prism 10.1.2. Gene pairs in which one gene showed zero expression across all tissues resulted in an undefined *r*. These pairs were labeled ‘undefined’ and excluded from the *r* distribution analysis. Tissue expression heatmaps of tandem- and segmental-derived genes were generated using the HeatMap Illustrator module in TBtools.

*Cis*-acting elements in the promoters of duplicated *CsCCR* genes were predicted using PlantCARE and classified into stress-, light-, ABA-, MeJA-, and total hormone-responsive modules. The element numbers in each module were summarized for each gene. To avoid non-independence caused by genes participating in multiple duplication events, a tandem-priority grouping strategy was adopted. Genes involved in any tandem duplication event were assigned to the tandem group, whereas genes involved only in segmental duplication were assigned to the segmental group.

### 4.8. Extraction of Total RNA and Synthesis of cDNA

Total RNA was isolated from the samples using the Plant Total RNA Extraction Kit (Proteinssci, Shanghai, China). RNA concentration and purity were determined with a NanoDrop ND-1000 spectrophotometer (Thermo Scientific, Waltham, MA, USA), while RNA integrity was confirmed by 1.2% agarose gel electrophoresis. Reverse transcription was performed using the HiScript II Reverse Transcription Kit (Vazyme, Nanjing, China) to synthesize cDNA. The obtained cDNA was diluted 12-fold with sterile ddH_2_O and stored at −20 °C for further RT-qPCR analysis.

### 4.9. RT-qPCR Detection and Gene Expression Analysis

The expression profiles of *CsCCR* genes across different tissues, abiotic stress treatments, and hormone treatments were examined using RT-qPCR to characterize their spatial and temporal expression patterns. Gene-specific RT-qPCR primers were designed using Primer Premier 5.0. *CsGAPDH* was selected as the internal reference gene based on previous reports in *C. sinensis*, where it was shown to exhibit moderate expression abundance suitable for RT-qPCR normalization [10,46,49]. All RT-qPCR primers for the genes were listed in Table S5. RT-qPCR was performed using the Bio-Rad CFX96 Real-Time System and Bio-Rad CFX Manager. A 20 μL reaction system was prepared using the Hieff^®^ qPCR SYBR Green Master Mix Kit (Yeason, Shanghai, China): 2 μL cDNA template, 10 μL SYBR Mix, 7.2 μL ddH_2_O, and 0.4 μL each of forward and reverse primers. The amplification protocol consisted of 40 cycles of initial denaturation at 95 °C for 5 min, denaturation at 95 °C for 5 s, and extension at 60 °C for 30 s. Melting curve analysis was performed using the instrument’s default parameters. Three biological replicates were analyzed, and the relative gene expression was calculated using the 2^−ΔΔCt^ method [50].

### 4.10. Data Processing and Analysis

Experimental data were organized using Microsoft Excel 2019, and figures were generated using GraphPad Prism 10.1.2. Statistical analysis of RT-qPCR data was performed using one-way analysis of variance (ANOVA) followed by Duncan’s multiple range test. Genomic count or distribution data were compared between groups using a two-tailed Mann–Whitney U test. Statistical significance was set at *p* < 0.05. All bar graphs were presented as the mean ± standard deviation (SD) of three biological replicates.

## 5. Conclusions

This study integrated comparative analysis across tea cultivars with a detailed investigation of ‘Shuchazao’. The results revealed a marked expansion of the *CCR* gene family in tea plants, driven by both WGD/segmental duplication and tandem duplication. Evidence from *Ka/Ks* ratios, expression correlation, and promoter *cis*-element differences indicated regulatory divergence among *CsCCR* genes derived from different duplication mechanisms. Integrating phylogenetic relationships, conserved motifs, cross-species synteny, and expression data indicated that *CsCCR3* was likely the main candidate gene involved in lignin biosynthesis in tea plants, and *CCR-like* genes such as *CsCCRL33* may function in auxiliary or compensatory roles in specific tissues or under stress conditions. This study provided a foundation for understanding *CCR* gene family expansion and potential functional specialization during the cultivation of perennial woody crops.

## Data Availability

Data are available upon reasonable request.

## Supplementary Materials

The following supporting information can be downloaded at: https://www.mdpi.com/article/10.3390/ijms27072957/s1.

## Abbreviations

ABA	Abscisic acid
CCR	Cinnamoyl-CoA reductase
CDD	Conserved Domain Database
CDS	Coding sequences
GA_3_	Gibberellin
HMM	Hidden Markov Model
*Ka/Ks*	Ratio of nonsynonymous substitutions per nonsynonymous site (*Ka*) to synonymous substitutions per synonymous site (*Ks*)
MCL	Markov cluster algorithm
PEG	Polyethylene glycol
RT-qPCR	Real-time quantitative reverse transcription PCR
UTR	Untranslated region
